# Cavernous Sinus Arteriovenous Fistula Presenting as Acute Third Nerve Palsy With Coexisting Sphenoid Sinusitis

**DOI:** 10.1002/oto2.70174

**Published:** 2025-10-23

**Authors:** Kuang‐Chien Chiang, Chung‐Wei Lee, Fu‐Ren Xiao, Chih‐Feng Lin

**Affiliations:** ^1^ Department of Medical Education National Taiwan University Hospital Taipei Taiwan; ^2^ Department of Medical Imaging National Taiwan University Hospital Taipei Taiwan; ^3^ Department of Neurosurgery National Taiwan University Hospital Taipei Taiwan; ^4^ Institute of Medical Device and Imaging National Taiwan University Taipei Taiwan; ^5^ Department of Otolaryngology, Head and Neck Surgery National Taiwan University Hospital Taipei Taiwan

**Keywords:** cavernous dural arteriovenous fistula, cerebral angiography, third nerve palsy

Isolated third nerve palsy may arise from various etiologies, ranging from benign microvascular ischemia to life‐threatening intracranial aneurysms.^1^ However, cavernous sinus dural arteriovenous fistula (CSdAVF) is a rare and often overlooked cause. We present an unusual case of CSdAVF manifesting as isolated third nerve palsy with pupillary involvement. Initial assessment and imaging suggested sphenoid sinusitis, but the patient's symptoms persisted after sinus surgery. This case highlights the importance of considering CSdAVF in the differential diagnosis of isolated oculomotor palsy, particularly when initial imaging reveals no compressive lesion.

## Case Report

**Figure 1:**
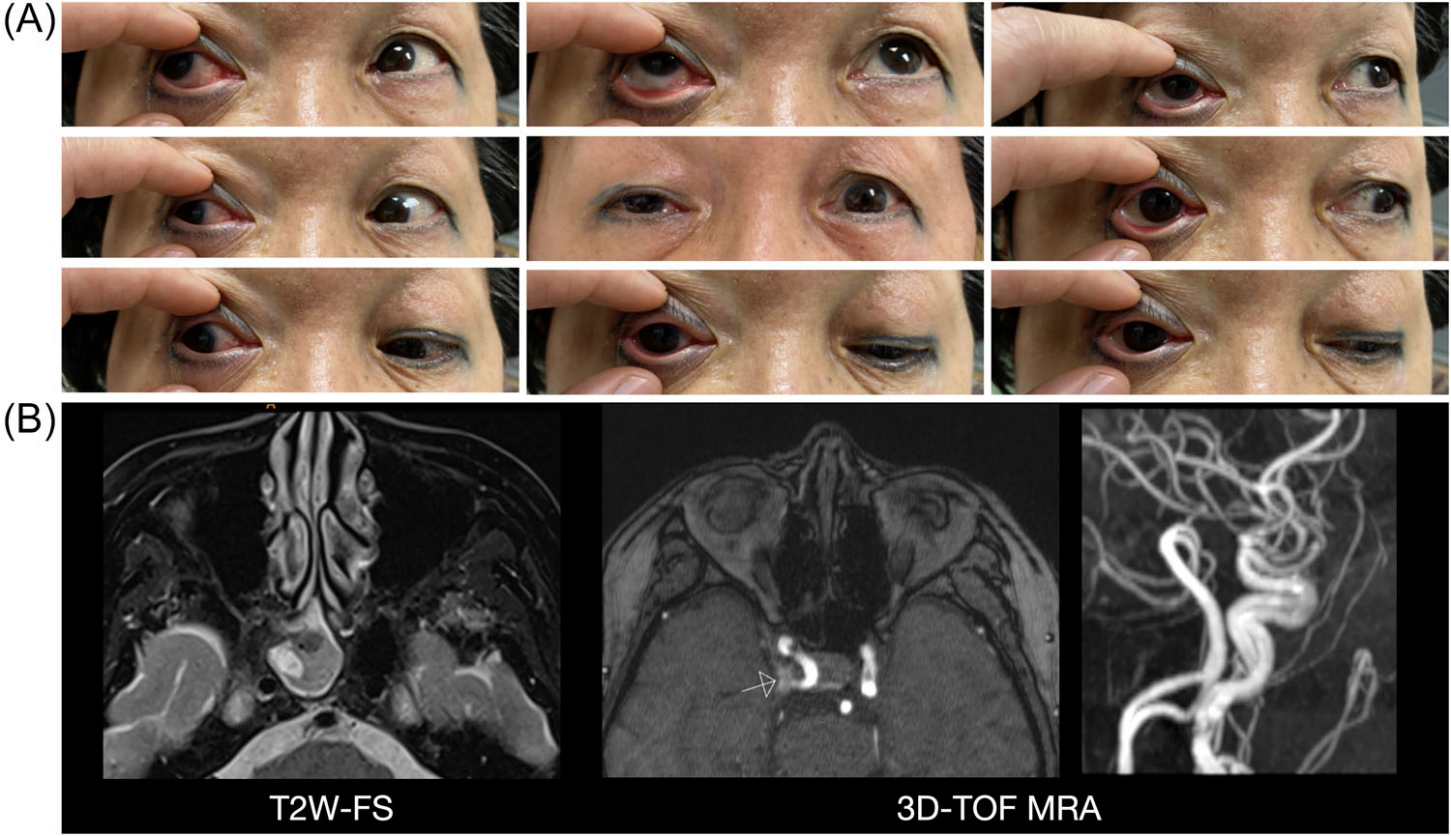
Initial presentation: right ptosis and limited eye movements (A). T2‐weighted fat‐saturated magnetic resonance imaging (T2W‐FS MRI) shows left sphenoid sinusitis (left). Three‐dimensional time‐of‐flight magnetic resonance angiography (3D‐TOF MRA) reveals focal signal changes near the right cavernous sinus (arrow, right) (B).

A 67‐year‐old woman presented to our emergency department with sudden‐onset right‐sided ptosis and diplopia for one day. Physical examination revealed right‐sided ptosis, mydriasis, and limited extraocular movements of the right eye with preserved abduction (Figure 1A). Binocular diplopia was present in all directions except right lateral gaze. Noncontrast brain computed tomography (CT) showed no intracranial hemorrhage. Contrast‐enhanced brain magnetic resonance imaging (MRI) and three‐dimensional time‐of‐flight (3D‐TOF) MR angiography (MRA) revealed no aneurysm, mass lesion, or destructive bony changes. However, right sphenoid sinusitis with focal signal changes in the right retrobulbar optic nerve and cavernous sinus (CS) was observed (Figure 1B). The initial differential diagnosis included optic neuritis and inflammation secondary to sphenoid sinusitis.

In the absence of blurred vision and decreased color vision, oculomotor palsy secondary to sinusitis was suspected. Endoscopic sinus surgery was performed, but the patient reported no improvement in ptosis and diplopia following the surgery.

Subsequent contrast‐enhanced MRA revealed early enhancement of the right CS, corresponding to the high signal region on 3D‐TOF MRA. Cerebral digital subtraction angiography (DSA) confirmed CSdAVF at the lateroposterior aspect of right CS (Supplemental Video S1, available online). Vessel supplies included the right internal maxillary artery, the middle meningeal artery, and bilateral meningohypophyseal trunks, with venous drainage into the right inferior petrosal vein and basilar plexus.

**Figure 2:**
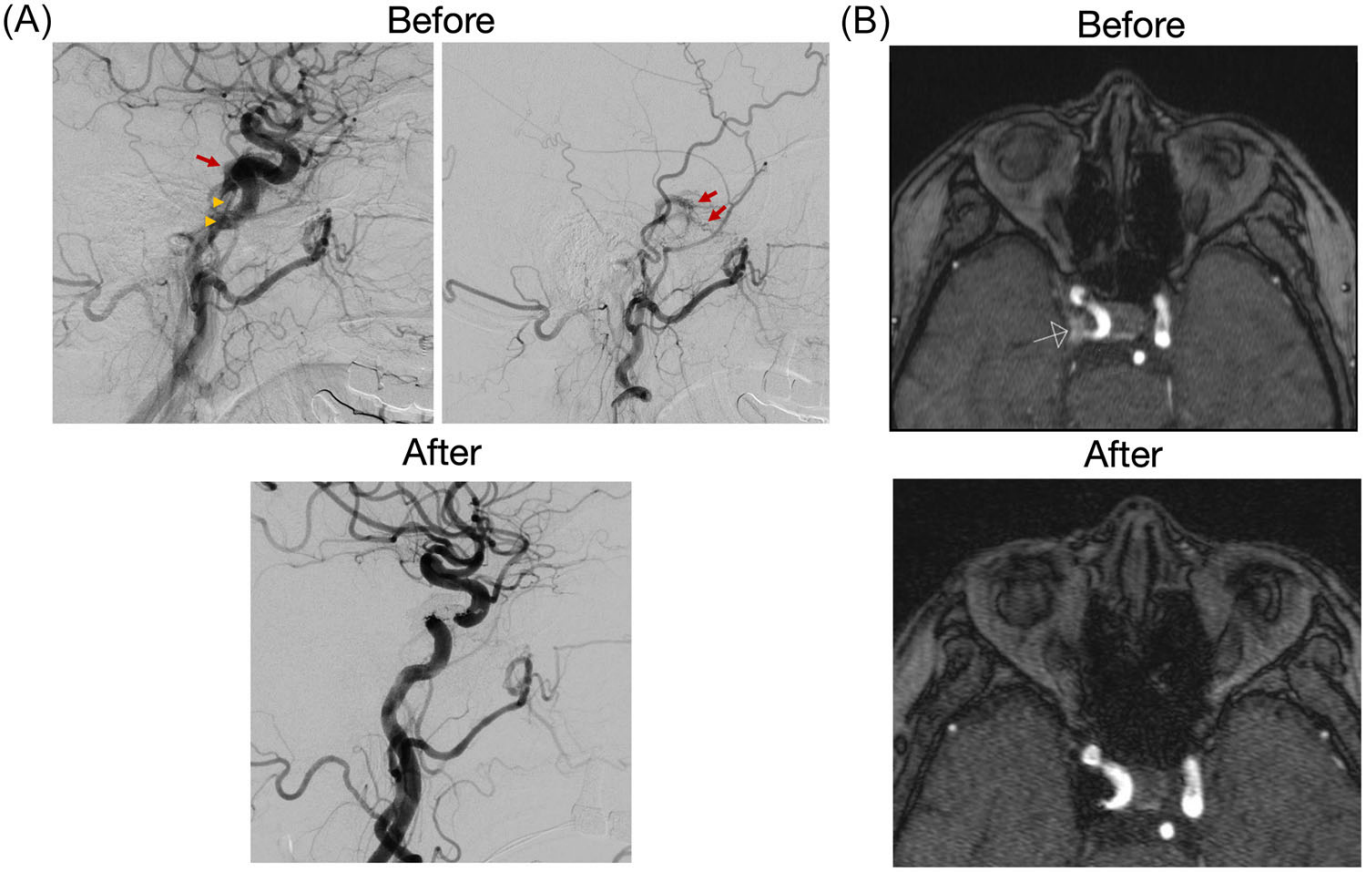
Angiography shows feeders from the right internal maxillary (red arrow, left) and middle meningeal arteries (red arrow, right), draining into the right inferior petrosal vein (arrowhead) (A). Post‐embolization magnetic resonance angiography (MRA) shows no residual fistula (B).

The patient underwent successful transvenous coil embolization (Figure 2A) and reported complete resolution of ptosis and diplopia afterwards. Follow‐up MRI 3 months later confirmed no residual CSdAVF (Figure 2B).

## Discussion

Acute third nerve palsy may result from lesions along its course, from the oculomotor nucleus in the midbrain to the levator palpebrae and extraocular muscles within the orbit.^1^, ^2^ While microvascular ischemia is the most common cause in patients over 50 years old, aneurysmal or neoplastic compression, cerebrovascular disease, infection, and inflammation should also be considered.^2^ Since pupillary fibers lie superficially within the oculomotor nerve, third nerve palsy with pupillary involvement raises suspicion for compressing causes, particularly a potentially life‐threatening intracranial aneurysm.^2^ However, in our case, the initial MRA revealed neither aneurysmal nor non‐aneurysmal compressive lesions. Our focus then shifted to the focal signal changes around the optic nerve, which were typically more suggestive of inflammation or infection.

The clinical presentation of CSdAVFs varies widely with flow dynamics and venous drainage pathways; similarly, imaging findings may range from unremarkable to prominent vascular dilatation.^3^ In retrospect, the signal changes at right CS on 3D‐TOF MRA were more associated with CSdAVF than with sinusitis. Nevertheless, the predominant drainage into the inferior petrosal sinus, along with the absence of superior ophthalmic or cortical vein engorgement, obscured the diagnosis. Given that delayed diagnosis may reduce the recovery potential of neurologic deficits,^3^ CSdAVF should be considered in patients with unexplained oculomotor nerve palsies. Importantly, unremarkable findings on noninvasive imaging do not reliably exclude the diagnosis.^3^

Notably, although DSA remains the gold standard for CSdAVF diagnosis, clinicians must carefully consider its invasiveness and potential complications.^4^ When the likelihood of CSdAVF is uncertain, a thorough review of the patient's history and prior noninvasive imaging should always precede the decision to pursue DSA.

The principal risk factors for CSdAVF encompass advanced age, female gender, venous thrombosis, hypertension, prior cranial trauma or surgery, and hypercoagulable states; however, in our patient, only advanced age and female gender are present as identifiable risks.

The hypothesis of isolated oculomotor palsy from sphenoid sinusitis remains uncertain. Such cases are reported,^5^ but abducens involvement is more common and usually accompanied by headache or orbital pain. Given the low likelihood of this mechanism and the invasiveness of surgery, CSdAVF should have been excluded before intervention.

This case report was exempted by the Institutional Review Board of National Taiwan University Hospital (registration number: 202503052W).

## Author Contributions

**Kuang‐Chien Chiang**, conceptualization, data curation, investigation, writing—original draft; **Chung‐Wei Lee**, data curation, methodology, resources, visualization; **Fu‐Ren Xiao**, funding acquisition, writing—review and editing; **Chih‐Feng Lin**, funding acquisition, writing—review and editing, resources, supervision.

## Disclosures

### Competing interests

The authors declare no conflicts of interest.

### Funding source

This work was supported by the National Taiwan University Hospital (No. 115‐M0010).

## Supporting information

Supporting Information.
